# Some Observations on Childhood

**Published:** 1916-06

**Authors:** E. S. Goodhue

**Affiliations:** The Doctorage, Hawaii


### Just Some Observations on Childhood.

BY E. S. GOODHUE, A. M., M. D., LL. D.

II.

Little sister's preference in literature is for poetry, and we have had to read to her a little from most of the poets, even Shakespeare and Milton.

Brother says that Milton is worse than algebra, but sister's eyes sparkle over the swing of the metre and the classical allusions.

As with other children, Longfellow, Stevenson, Field and Riley are favorites, but sister has tastes of her own, and is fond of many poems which would not seem to appeal to her. These she has learned to repeat with a good deal of expression. Then, in certain moods, she takes her book and "reads" her own poetry out of it, asking me to write down what she says after it is "all sentenced."

At first her "poetry" was only a jingle, as when she was two years and three months:

"I got a trout
Without a doubt."

From this time until she was four years old she could or would not sound the letter "l" but used "n" instead, acquiring quite a knack of sounds like "f-n ew" for flew, "c-noud" for cloud.

It was "I nove you-ten me truny," and so on.

I have not been able to trace this odd inability to use the letter "l" to any cause; possibly it is an atavism.

The day she made up the trout verse she composed:

"There was a boy who walked in the snow,
He had legs to step with
And legs to grow."

A year later:

"A little bird flew up and kissed God
And came back with a piece of cloud in his bill."

Fancies like these were common with her, and when she had what she felt was a good idea she was not so particular about the metre. I told her that the last verse quoted wasn't very good poetry, but she said it had better be put down, as there was a "lot of sense in it!"

Some of the things she named "Queerilies" were:

"I will throw stones at the moon."

"I'd like to have a real live person to *slap.*"

"I wish an eagle would come down and take me to its nest
*And eat me up.*"

"I'd like to be such a good woman that little girls would like to come and stay with me."

Near Christmas she told me a story ending with—

"The Christmas tree jumped for joy,
The moon and stars came down to see."

"Papa," she said to me one day after my failure to answer a question, "You don't seem to know so very much. If you read your Bible you will learn wisdom."

Who can unravel a child's thoughts or inspirations? As sister sat "playing" the piano one day, she jumped up with enthusiasm and asked—

"Is there another world than this, papa?"

"Yes," I answered, "in due time there will be another."

"Where is it?" abruptly.

"Up in heaven, I suppose," I answered.

"Oh," she said, and ran back to her improvisations.

August 7th, fourth year:

"The flowers grow in my garden so sweet,
But no one comes to pick them, my love."

November 30th, same year, standing under the pines talking partly to herself and partly to me, in cadence:

"The pine trees are talking to each other,
That tree is so fresh and sweet—
The mountain changes its color,
Now it is going to bed wrapped in its clouds—
Yesterday was so clear and bright, too—
Oh, I love to be out."

March 6th, fifth year, watching steam from the kettle:

"It's the hot water's breath."

Reading aloud:

"If you keep what you've got
You'll always have what you want."

In the evening, after doing some of her own "composing," she said to her mother: "I'm making poetical dough, mother."

May 25th, same year. By herself, looking out of the south window at the stars:

"O glad darkness and beautiful stars scattering seeds of light!
God is looking down and watching, always watching.
Nothing will hurt you under his care.
O Mother Moon!
Counting her school children, the stars
O darkness! I could hug you sweetly,
All the stars are children cuddling together,
O! they make me think of many other things,
O! glad stars! O Night! O Moon!"

June 7th, sixth year, singing from book:

"Shadows are in the clouds,
I could see them gentle figures make,
I cannot tell you what they are
So many, many there are."

June 1st, same year, after seeing a rainbow:

"The rainbow's colors took my heart away from me."

Little sister has always possessed a joyful spirit, as most normal children do, and in the last year has shown a keen appreciation of fun.

Brother, too, by this time has a pretty well developed sense of humor, and will stand Mark Twain by the hour if some one will read to him and laugh with him.

November 18th, seventh year. Sister's mother said to her:

"Don't touch anything—the man has just put everything away under the house."

"Oh," answered sister, gaily, "the sky and the moon and the stars—yes, *everything*, mamma!"

When she and brother came to the cane field:

"Now we're in the land of Canaan, brother."

Shortly after she came in with a conundrum:

"When wouldn't the earth have anything to chop with? When it's turning on its axes."

"What a place for swans the Suanee river must be!"

December 16th, same year, after reading to her about the influence "Uncle Tom's Cabin" had in the abolition of slavery, she remarked:

"Well, the women have done as much by their words as the men who went to do the things—haven't they?"

And—

"I wouldn't like to be an old maid, because they would say, 'O there's an old maid.' I would rather be a widow, for then they would pity me."

A few days ago she came in all enthusiasm:

"I've seen all these things and made a verse about them. Put it down quick. It's called 'A Short Chorus.'"

"Our roses fair are blooming,
Our house-finches are singing,
Our gobbler is gobbling,
Our turkey hen is calling,
Our little turkeys peeping,

And they make quite a band, too,
That's good enough for you,
With a mynah bird or two—
To join it, *I bet you.*"

And yesterday:

"Jeremy Stickles
Got into the pickles
And smeared them all over his face;
As soon as he was able,
He went to the table
To hear his mother say grace."

Sister has a very deep interest in writers. She wants to know all about them, but cares nothing about kings, lords or princes. She asks me to write to different ones for their opinions about things, and it is not always easy to persuade her that I cannot well do it. But she has several letters she prizes highly, from Mark Twain, Mrs. Howe, Laura E. Richards, Weir Mitchell, Roy Stonnard Baker, Jack Lardon and others.

Now I think that in the education of these children, the definite schooling to follow home training and influence is pretty well indicated by the individual tastes manifested. We know what not to expect from them, anyhow.

It would be a crime to try to make a college professor of brother, and to refuse sister a liberal education would be equally unjust. So with every child there are means of ascertaining the natural bent, especially if it has not been forced into artificial channels; if it has been allowed to develop according to nature, guarded, it is true, and, in a measure directed, but never "shocked or strained," always in an intellectual sense, at least, permitted to pass in the *locus minoris resistenciae.*

"A boy in the field," says Dr. Bridge, "or on the sea, may in four years learn of his fellows and those about him, as many things such as they are, as he would acquire at school, and be more vigorous with each successive month. Every child has the natural right to the most successful and complete career in life that is possible to it."

The Doctorage, Hawaii, January, 1916.

